# Damage Identification of Railway Bridges through Temporal Autoregressive Modeling

**DOI:** 10.3390/s23218830

**Published:** 2023-10-30

**Authors:** Stefano Anastasia, Enrique García-Macías, Filippo Ubertini, Vincenzo Gattulli, Salvador Ivorra

**Affiliations:** 1 Department of Civil Engineering, University of Alicante, Carr. de San Vicente del Raspeig sn, 03690 Alicante, Spain; stefano.a@ua.es (S.A.); sivorra@ua.es (S.I.); 2 Department of Structural Mechanics and Hydraulic Engineering, University of Granada, C/ Dr. Severo Ochoa s/n, 18071 Granada, Spain; enriquegm@ugr.es; 3 Department of Civil and Environmental Engineering, University of Perugia, 06100 Perugia, Italy; 4 Department of Structural and Geotechnical Engineering, Sapienza University of Rome, Via Eudossiana Nr. 18, 00184 Rome, Italy; vincenzo.gattulli@uniroma1.it

**Keywords:** autoregressive modeling, damage identification, moving loads, SHM, statistical pattern recognition, railway bridges

## Abstract

The damage identification of railway bridges poses a formidable challenge given the large variability in the environmental and operational conditions that such structures are subjected to along their lifespan. To address this challenge, this paper proposes a novel damage identification approach exploiting continuously extracted time series of autoregressive (AR) coefficients from strain data with moving train loads as highly sensitive damage features. Through a statistical pattern recognition algorithm involving data clustering and quality control charts, the proposed approach offers a set of sensor-level damage indicators with damage detection, quantification, and localization capabilities. The effectiveness of the developed approach is appraised through two case studies, involving a theoretical simply supported beam and a real-world in-operation railway bridge. The latter corresponds to the Mascarat Viaduct, a 20th century historical steel truss railway bridge that remains active in TRAM line 9 in the province of Alicante, Spain. A detailed 3D finite element model (FEM) of the viaduct was defined and experimentally validated. On this basis, an extensive synthetic dataset was constructed accounting for both environmental and operational conditions, as well as a variety of damage scenarios of increasing severity. Overall, the presented results and discussion evidence the superior performance of strain measurements over acceleration, offering great potential for unsupervised damage detection with full damage identification capabilities (detection, quantification, and localization).

## 1. Introduction

The railway plays a pivotal role in Europe’s ambitions toward a more sustainable and carbon-neutral transportation system. Among the strategic goals identified in the White Paper on Transport released in March 2011 [1], it is projected that by 2030 and 2050, respectively, 30% and 50% of the road freight spanning over 300 km will transition to alternative modes less dependent on fossil fuels, such as rail or waterborne transport. To this end, it is imperative to rely on a robust and efficient infrastructure capable of accommodating the increased demand resulting from this modal shift. In this context, bridges represent critical assets in the railway infrastructure. In Europe, 35% of the more than 300,000 railway bridges spread across 200,000 km of railways are over 100 years old [2,3]. As a result, the degradation over time of these structures, which often surpass their life expectancy, poses a high risk to the overall integrity of the railway network. Indeed, maintenance and renewal costs experience rising trends exceeding EUR 25 billion annually [4]. This has spurred vast research on structural health monitoring (SHM) as an efficient strategy to reduce inspection and maintenance costs, mitigate downtime periods, and extend the lifespan of structures. Nonetheless, despite the evident benefits of SHM, its routine integration into the civil engineering sector remains limited [5]. Among the factors contributing to the slow technology transfer of SHM, the lack of easily implementable and interpretable damage identification algorithms as well as the reduced number of real-world demonstration examples stand out [6].

The SHM paradigm advocates the utilization of long-term non-destructive monitoring to complement traditional inspections with quantitative information. This enables the optimization of maintenance and conservation interventions by providing a prompt identification of structural pathologies [7,8,9]. Driven by decreasing costs and advancements in sensing and data acquisition technologies, SHM strategies are shifting toward the implementation of heterogeneous and dense sensor networks capable of offering comprehensive health assessments [10,11,12]. A large variety of instrumentation strategies have been reported in the literature, from more classical wired sensors installed on the structure, Internet-of-Things (IoT) sensors [13], to more innovative in-vehicle monitoring approaches [14,15]. In the realm of railway bridge SHM, a broad variety of electrical measurement and sensing technologies are available, including strain gauges, accelerometers, weigh-in-motion sensors, titlmeters, acoustic emission sensors, digital image correlation (DIC), and geophones [16,17,18], among others. The implementation of fiber optic sensors (FOs) has witnessed a significant increase in recent years due to their numerous advantages over conventional sensors. Some of these advantages include high sensitivity, immunity to electromagnetic interference, ability to function in harsh environments, and potential for measuring strain, temperature, and vibration [19]. Technologies like fiber Bragg grating (FBG) as well as Fabry–Perot and Mach–Zehnder interferometers, which exploit the principles of optical reflectometry and interferometry, also allow for multiplexing and distributed strain sensing. In railway bridge SHM, FBG sensors are frequently used for quasi-distributed strain measurements by using numerous gratings at discrete locations along the fiber. These configurations provide high flexibility to monitor the strain profile along any desired geometry [20,21]. In addition, given their direct correlation with stresses and deflections, strain measurements can act as direct damage-sensitive features, providing a clear marker of the safety, functionality, and performance of structures [22]. For a comprehensive state-of-the-art review of the implementation of FOs for SHM of civil engineering structures, interested readers may refer to references [23,24].

In general terms, structural damage is defined as an alteration in the material and/or geometric properties of structural members, as well as changes in the boundary conditions, with respect to the initial (healthy) reference condition [25]. To identify such pathologies, the process of damage identification can be divided into the extraction of damage-sensitive features, data normalization (elimination of environmental/operational effects), and damage classification [26]. The first step involves extracting certain damage-sensitive features from the monitoring data. In civil engineering, considerable efforts have been devoted to assessing modal properties through ambient vibration testing and operational modal analysis (OMA). The damage-sensitive nature of modal features (i.e., resonant frequencies, mode shapes, and damping ratios) relies on their dependence on the intrinsic stiffness and mass properties of structures [27,28,29]. Nonetheless, while these approaches are highly effective for global damage identification [30,31], their effectiveness in identifying local defects is limited. On the one hand, their potential for identifying local pathologies is largely contingent on the ability to identify high-frequency modes, which is particularly challenging in real-world structures under ambient vibration conditions [32]. On the other hand, OMA-based techniques typically operate with small-magnitude white random excitations, which fail to excite non-linearities that might reveal damage more conspicuously.

Time-series modeling can directly operate on transient signals without assuming Gaussianity, which provides a more flexible framework to address damage identification in railway bridges [33,34]. Various time-series modeling techniques are often employed in SHM, including wavelet transform [35], Hilbert transform [36], Bayesian Fast-Fourier transform [37], and Cepstral coefficients [38]. Feature extraction methods based on autoregressive (AR) modeling have proved particularly efficient and relatively straightforward to implement [39]. These techniques, along with their variations, such as AR models with exogenous inputs (ARX) and AR models with moving average (ARMA), regress the structural response data onto past values [40]. On this basis, a variety of damage-sensitive features have been utilized in the literature, including model residual variance, AR parameter vectors, and AR model spectra [41]. In this context, it is worth highlighting the work of Meixedo and co-authors [42], who developed a damage identification approach based on novelty analysis of continuously acquired series of AR coefficients from acceleration records for passing trains. The effectiveness of the proposed approach was demonstrated through an application example of a high-fidelity digital twin of the Sado railway bridge in Portugal, reaching minimal false detection incidences of 2%. A similar study was recently reported by Wang et al. [43], who investigated the use of strain measurements and the residual errors of an AR model to identify damage in high-speed railway box girders. Although their work demonstrated the potential of strain measurements for damage identification, it did not include a comparison with the results obtained using acceleration data, nor did it investigate the influence of environmental factors. Building upon these successful implementations, the use of AR modeling and FOs for dynamic strain sensing may offer an ideal framework for the development of new SHM systems with superior damage identification capabilities. Nonetheless, the use of dynamic strain sensors and time-series modeling for damage identification of railway bridges remains poorly explored in the literature.

In this light, this work explores for the first time in the literature the use of AR modeling to continuously extract damage-sensitive features from deformation measurements under varying environmental/operational conditions (EOCs) and compare their effectiveness for damage identification against acceleration data. The investigation is framed within a research project dedicated to the SHM of a real-world, in-operation steel truss railway bridge—the Mascarat Viaduct in Alicante (Spain). With the aim of assessing the effectiveness of the last rehabilitation interventions conducted on the bridge in 2016, the viaduct was instrumented with a dense network of deformation sensors and accelerometers. Some of the acquired recordings, captured both during the passage of trains and under ambient excitation conditions, were retrieved for analysis in this work. On this basis, a detailed three-dimensional finite element model (FEM) of the viaduct has been constructed and experimentally validated. Subsequently, an extensive synthetic database has been generated, considering the influence of environmental (temperature) and operational (train’s speed and weight) factors on the deformations and accelerations experienced by the viaduct. Additionally, the developed FEM has been employed to simulate various damage scenarios. The generated monitoring database was used to investigate the efficacy of an SHM system that leverages the continuous extraction of AR coefficients from strain data during train passages as damage-sensitive features. To minimize the effects of EOCs, an innovative statistical pattern recognition approach combining density-based spatial clustering of applications with noise (DBSCAN) and principal component analysis (PCA) is presented. The reported numerical results demonstrate the superior performance of strain data over acceleration in achieving prompt identification of early-stage damage.

The remainder of this paper is organized as follows. Section 2 presents the theoretical fundamentals of the proposed approach. Section 3 presents the investigated case studies and the obtained numerical results and discussion. Finally, Section 4 closes the paper with some concluding remarks.

## 2. Theoretical Framework

**Figure 1:**
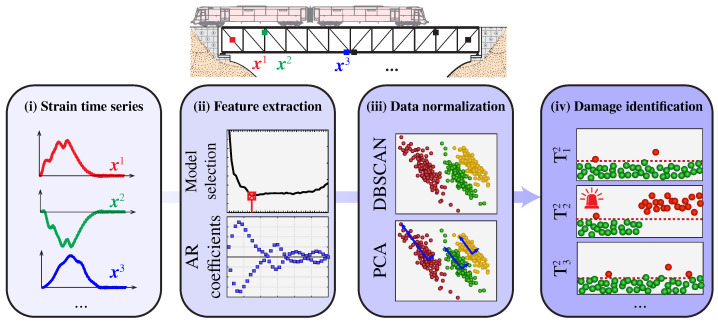
Flowchart of the proposed damage identification approach leveraging the continuous extraction of AR coefficients from strain data.

The damage identification approach proposed in this work is outlined in Figure 1. The methodology involves four consecutive steps for assessing damage in railway bridges instrumented with a long-term network of strain sensors. Firstly, during the passage of a train across the bridge, strain time series are recorded and pre-processed (i). To trigger the data acquisition, a variety of low-cost sensors are available in the market, including microelectronic mechanical systems (MEMSs) accelerometers, velocimeters, ultrasonic sensors, and closed-circuit television (CCTV) cameras. Afterward, the recorded strain data are utilized to extract a set of sensor-level AR coefficients as damage-sensitive features (ii). Subsequently, a two-step data normalization approach (iii) is employed to mitigate the effects of EOCs (primarily environmental temperature and train speeds/loads). This process comprises two stages: the application of a clustering approach to identify different train speeds/loads followed by cluster-wise statistical pattern recognition. For this purpose, DBSCAN and PCA were adopted in this work. Finally, damage identification is accomplished through novelty analysis of the cleansed AR coefficients using a set of sensor-based control charts (iv). In the remainder of this section, the theoretical details of each step in the proposed methodology are concisely overviewed.

### 2.1. Feature Extraction: Autoregressive Modeling

Discrete response time-series data $x_{j}$, j=1,…,n, can be expressed by an *m*-order AR(*m*) model as:

(1) $$x_{j}=\sum_{i=1}^{m}a_{i}\;x_{j-i}+\varepsilon_{j},$$

in such a way that $x_{j}$ is defined as a linear combination of the *m* previous response values multiplied by the AR parameters $a_{i}$ plus a residual error term $\varepsilon_{j}$. The AR model in Equation (1) can be cast in matrix form as:

(2) $$\left[\begin{array}{c} x_{m+1}\\ x_{m+2}\\ \vdots \\ x_{n} \end{array}\right]=\left[\begin{array}{cccc} x_{1} & x_{2} & \ldots & x_{m}\\ x_{2} & x_{3} & \ldots & x_{m+1}\\ \vdots & \ddots & \ldots & \\ x_{n-m} & \ldots & \ldots & x_{n-1} \end{array}\right]\left[\begin{array}{c} a_{m}\\ a_{m-1}\\ \vdots \\ a_{1} \end{array}\right]+\left[\begin{array}{c} \varepsilon_{m+1}\\ \varepsilon_{m+2}\\ \vdots \\ \varepsilon_{n} \end{array}\right].$$

The AR coefficients $a_{i}$ can be estimated by solving the overdetermined (m>n) set of equations in (2) through the least-squares method or the Yule–Walker approach [44]. Given that the AR coefficients are determined by the stiffness/mass properties of the structure, the time series of continuously extracted $a_{i}$ coefficients are employed in this work as damage-sensitive features.

The performance of AR models critically hinges on the model order, *m*, which represents the number of past observations used for predicting the current value of the time series. Generally, an excessively high parametrization of the model tends to overfit noise and lose generality. Conversely, an excessively low order fails to accurately capture the underlying physical mechanisms governing the investigated system [45]. Additionally, the model order dictates the dimensions of the observation matrix in Equation (2) and, therefore, it also determines the computational workload in the feature extraction process. Since the model order is typically unknown beforehand, it is commonly determined through parametric analyses evaluating a certain error metric [46,47]. Among the variety of metrics available in the literature, the Akaike information criterion (AIC) and the Bayesian information criterion (BIC) are notably efficient in achieving a balance between prediction accuracy and model complexity. The BIC and AIC metrics are defined as follows [48,49]:

(3) $$BIC\left(m\right)=n\ln\frac{RSS(m)}{n}+m\ln n,\;AIC\left(m\right)=n\ln\frac{RSS(m)}{n}+2m,$$

where RSS(m) represents the sum of squared residuals of the AR model of order *m*, that is:

(4) $$RSS\left(m\right)=\sum_{i=m+1}^{n}{\left(y_{i}-{\hat{y}}_{i}\left(m\right)\right)}^{2},$$

with $y_{i}$ and ${\hat{y}}_{i}\left(m\right)$ being the measured data and the estimations of the AR(*m*) model, respectively.

Considering any of these metrics, different AR models are constructed for a given acceleration/strain record considering increasing model orders. Subsequently, the error metrics are estimated for all the models and represented in an error-versus-model-order curve. These curves often display a distinct elbow, which can be used to identify the optimal model order. Alternatively, the optimal model can be identified as the one with the minimum error. In Section 3, we delve into a detailed discussion regarding the implications of choosing between these two approaches.

### 2.2. Elimination of Operational/Environmental Effects and Anomaly Detection

Extensive literature underscores the significant impact of diverse EOCs on the static and dynamic responses of civil engineering structures. These factors affect the boundary conditions and the mass/stiffness characteristics of structures, giving rise to important in fluctuations in their behavior across varying spatial and temporal scales (refer, e.g., to [39,50]). In the context of this study, the AR coefficients may also be notably influenced by EOCs such as train loads, train speeds, temperature, and humidity. This translates into benign fluctuations in the AR coefficients, which can mask the appearance of structural damage. Hence, in order to attain efficient damage identification, it becomes paramount to adopt a certain statistical pattern recognition algorithm capable of discerning between EOC- and damage-induced anomalies. This is conducted in this work by the combination of a clustering model and a statistical pattern recognition algorithm.

In broad terms, statistical models for data normalization aim to reproduce an observation matrix denoted as $Y\in R^{N\times m}$, which comprises *m* damage-sensitive features and *N* observations. In this work, Y is composed of time series data representing the AR(m) coefficients extracted from the strain measurements recorded by a specific sensor. Once trained, the predictions of the model $\hat{Y}$ can be used to mitigate the variance in Y caused by EOCs, forming the so-called residual error matrix $E=Y-\hat{Y}$. On this basis, should damage develop, this solely impacts Y, while matrix $\hat{Y}$ remains unchanged. Consequently, matrix E only contains modeling inaccuracies and any anomalies induced by damage. This makes matrix E suitable for use in damage identification. The statistical model requires training with a set of $t_{p}$ feature samples defining a baseline in-control population, often referred to as the training period. As a standard, one year is commonly selected to encompass both the daily and seasonal fluctuations the structure may undergo.

#### 2.2.1. Data Clustering Using DBSCAN

To automatically identify sets of AR coefficients extracted for varying train loads, the DBSCAN clustering algorithm was adopted in this work. This algorithm has been chosen due to its capability to define clusters within dense connected regions of data, a characteristic observed when analyzing AR coefficients extracted for different train configurations (weight and speed). Readers can find a comprehensive description of the DBSCAN algorithm in the original work by Ester and co-authors [51]. Here, we present only the fundamental steps of the algorithm:

1. For each as-yet unlabeled data point *p* within the training period $Y_{t_{p}}$, consider the points located within a region of radius ϵ as per the Euclidean distance (ϵ-neighborhood).
2. If at least MinPts points (including *p*) fall within the distance ϵ from *p*, a new cluster *C* is defined. Subsequently, *p* is designated as a *core point* of *C*, while the remaining MinPts−1 points are marked as *directly reachable* from *p*. If this condition is not met, *p* is labeled as a *noise point*.
3. The cluster *C* is expanded to encompass all points indirectly reachable from *p*. A point *q* is considered reachable if there exists a path of directly reachable points between *p* and *q*.
4. If point *q* is not directly connected to any other point, it is identified as a *border point*.
5. Once all paths originating from *p* terminate at a border point, the next unlabeled point is processed until none remain.

The performance of the DBSCAN algorithm is determined by two user-defined input parameters: MinPts and ϵ. In this study, MinPts was manually specified after inspecting the monitoring data. Instead, the optimal value for the neighborhood size parameter ϵ was automatically determined using a k-distance graph. This graph plots the distances to the k-nearest neighbors in descending order [52], often resulting in a curve with a noticeable elbow. The value associated with this elbow can be regarded as the optimal setting for the search distance. Finally, it is important to remark that the clustering of the AR coefficients was exclusively carried out within the training period. Subsequently, during the damage assessment phase, data points were assigned to previously identified clusters based on the minimum Mahalanobis distance.

#### 2.2.2. Cluster-Based Data Normalization Using PCA

Once the AR coefficients are clustered according to different train load configurations, the remaining variance induced by environmental factors can be minimized by employing a data normalization technique. In cases where environmental data are available, and input–output techniques such as multiple linear regression (MLR) [53] may be adopted. Nonetheless, this approach requires specific environmental sensors and, subsequently, increases the data storage demands. Therefore, for the sake of generality, PCA was adopted in this work as an input-only data normalization approach. PCA is a dimensionality-reduction technique utilized to transform databases into lower dimensional subspaces without significant loss in data variance [54]. Mathematically, PCA is defined as an orthogonal linear transformation that reorients data to a new coordinate system where the first principal component (PC) holds the highest variance, the second PC holds the second-highest variance, and so forth. These PCs correspond to the eigenvectors of the covariance matrix of the original data, forming an orthogonal basis of uncorrelated components. Let $Y_{n}$ denote the normalized version (i.e., zero mean and unit variance) of the observation matrix within the training period and pertaining to a certain cluster *C*, $Y_{t_{p},C}$. The PCs can be obtained by the eigen-decomposition of the covariance matrix $\mathrm{Cov}\left(Y_{t_{p},C}\right)$ as:

(5) $$\mathrm{Cov}\left(Y_{t_{p},C}\right)U=US^{2},$$

where the eigenvectors in the columns of U (loading matrix) represent the PCs, and the eigenvalues constitute the diagonal terms of $S^{2}$. The PCs are arranged in descending order according to the diagonal terms of $S^{2}$, which indicate the proportion of the total variance in $Y_{t_{p},C}$ explained by the PCs [55]. The transformed data matrix T (scores matrix) is formed by projecting the original data in $Y_{n}$ over the space spanned by the PCs in U:

(6) $$T=Y_{n}U.$$

In the realm of SHM, the first PCs retain the most significant contributions to the variance in the features matrix $Y_{t_{p},C}$ induced by EOCs, whereas variations associated with noise and feature extraction inaccuracies are contained in the latter components [32]. In this light, matrix $Y_{n}$ can be approximated by mapping back a reduced subset of PCs onto the original data space. Specifically, if only the first *l* columns of matrix U are collected into a reduced matrix $U_{l}$, matrix $Y_{n}$ can be obtained as:

(7) $${\hat{Y}}_{n}=Y_{n}\left(U_{l}\;U_{l}^{T}\right).$$

Note that the loading matrix is determined solely from data within the training data and remains constant when applying the prediction expression in Equation (7). It is also important to highlight that the effectiveness of PCA as a data normalization technique is largely determined by the number of retained PCs, *l*. If this dimension is too small, part of the EOCs will not be properly captured. Conversely, an excessively large number of retained PCs can lead to over-fitting and the subsequent loss of generality in the model. A common rule of thumb in the SHM literature is to choose *l* PCs explaining more than 80% of the cumulative variance [42,56].

#### 2.2.3. Novelty Analysis Using Sensor-Based Control Charts

Novelty analysis and statistical process control charts are popular unsupervised tools for detecting damage-induced anomalies within the time series of residuals in E. Specifically, control charts display a certain statistical measure over time, reflecting deviations in the distribution of residuals compared to the training period. This facilitates the identification of performance anomalies (potentially induced by damage) in the shape of accumulations of data points exceeding certain thresholds or in-control regions.

Among the diverse range of control charts available in the literature, Hotelling’s $T^{2}$ control chart [57] stands out as one of the most commonly employed methods in the field of SHM. This control chart displays the $T^{2}$ statistic (squared Mahalanobis distance), which is defined as:

(8) $$T_{i}^{2}=r{\left(\bar{E}-\bar{\bar{E}}\right)}^{T}\Sigma_{0}^{-1}\left(\bar{E}-\bar{\bar{E}}\right),\;i=1,2,\ldots ,N/r,$$

where the parameter $r\in N^{*}$ represents the subgroup size. The term $\bar{E}$ refers to the mean of the residuals within the subgroup comprising the last *r* observations, while $\bar{\bar{E}}$ and $\Sigma_{0}$ are the mean values and covariance matrix of the residuals during the training period, respectively. To classify the data points as in-control (undamaged) or out-of-control (damaged), an upper control limit (UCL) associated with a 1−α confidence level must be defined. It is important to emphasize the importance of setting proper thresholds for damage detection to keep minimal false alarm rates while maximizing the detection of actual damage states [58]. In this work, the UCL was determined in a frequentist fashion by analyzing the residuals over the training period. Nevertheless, more sophisticated approaches may be adopted such as the theoretical F-distribution of Hotelling’s control chart, assuming the residuals follow a multivariate Gaussian distribution [10], or through receiver operating characteristic (ROC) curves [31]. Once the UCL is set up, it is important to note that control charts primarily serve as Level-I damage classifiers (i.e., non-damaged or damaged). Nonetheless, in this work, sensor-level control charts are defined for the AR coefficients extracted from all the sensors in the SHM system. Consequently, higher damage identification levels (localization and quantification) can be achieved by analyzing the anomalies identified across the various sensors within the system.

## 3. Numerical Results and Discussion

The effectiveness of the previously introduced damage identification approach was appraised through two case studies: (i) a theoretical example involving a simple supported beam for moving loads and (ii) a real-world steel truss railway bridge. The first case study is intended to explore the benefits of adopting strain measurements for SHM, while the second one is aimed at demonstrating the effectiveness of the proposed approach in a real railway bridge under realistic operational conditions.

### 3.1. Case Study I: Simply Supported Bridge

**Figure 2:**
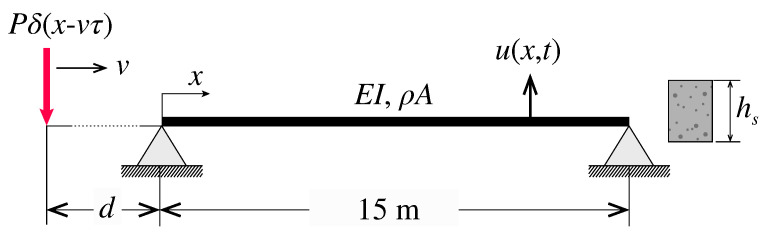
Simply supported Euler–Bernoulli beam subjected to a single moving point force.

The first case study is depicted in Figure 2 and corresponds to a bridge configuration analyzed by the Spanish standard for railway bridges IAPF-07 [59]. This configuration consists of a simply supported Euler–Bernoulli beam of length L=15 m, constant mass per unit length ρA=15 t/m, flexural stiffness EI=7694.081 MPa, and constant modal damping ratio ζ=2%. Without loss of generality, let us assume the beam has a rectangular cross-section of height $h_{s}$. A single point moving load *P* traverses the bridge at a constant speed *v*, and it is initially located at a distance *d* from the origin. Mathematically, such a load can be written as p(x,t)=Pδ(x−vt−d), with δ denoting the the Dirac delta function. The main interest of this case study is that the related moving load problem for any train load configuration can be solved in analytical terms. The closed-form solutions in terms of vertical accelerations and normal strains are reported in Appendix A. In the subsequent analyses, the 56-axle ICE2 train was considered (the train composition is given in [59]), and the dynamic response was extracted considering the first ten modes of vibration with resonant frequencies in the broadband up to 80 Hz.

**Figure 3:**
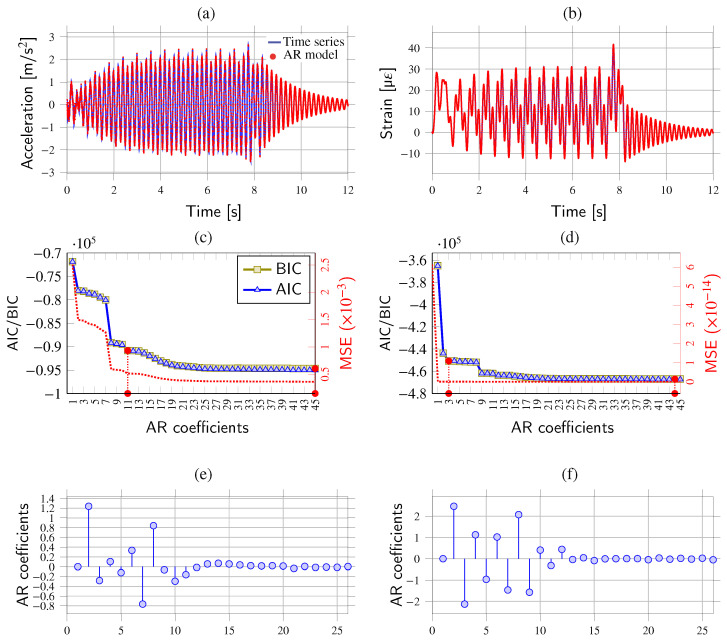
Acceleration (**a**) and strain (top surface) (**b**) time series and AR predictions at mid-span of a simply supported beam traversed by the ICE2 train traveling at 160 km/h (Δt=1 ms). Quality parametric analyses for increasing model orders for AR modeling of the acceleration (**c**) and strain (**d**) time series (red filled dots represent the optimal model orders determined as the minimum and elbow points of the BIC curve). AR coefficients (m=26) fitted using the acceleration (**e**,**f**) strain time series.

The estimated acceleration and strain (top fiber) time series at the mid-span of the beam are depicted in Figure 3a,b, respectively. These time series are used to determine the optimal AR models through parametric analyses, considering increasing orders ranging from m=1 to m=45, as shown in Figure 3c,d. We considered three different quality metrics: BIC, AIC, and mean squared error (MSE). The optimal model order is determined by selecting the model with the lowest error metric or, alternatively, by identifying the point where the change in the chosen metric stabilizes, often referred to as the elbow point. Figure 3e,f show the AR coefficients obtained for model orders of m=26 for both the acceleration and the strain time series.

Figure 3c,d reveal that AIC and BIC metrics exhibit similar behavior, while the MSE metric converges at lower model orders. These findings indicate that the acceleration time series requires significantly higher AR model orders compared to the strain series. The reason is that the strain time series is less affected by fast-oscillation components, requiring lower model orders to achieve comparable prediction accuracy. Notably, the BIC curves show elbow points at model orders of m=11 for acceleration and m=3 for strain. The corresponding predictions from the optimal AR models are also presented in Figure 3a,b with red dot markers, demonstrating very close fits to the original time series in both cases. In conclusion, these findings indicate that low-order AR models are effective in approximating strain measurements, yielding a more compact set of AR coefficients well-suited for the purpose of damage detection.

**Figure 4:**
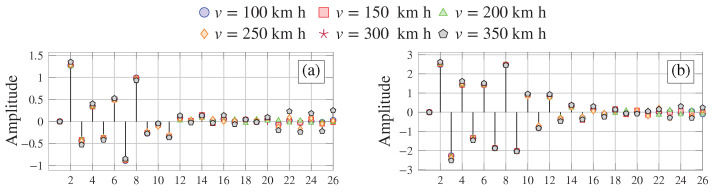
Parametric analysis of AR coefficients extracted from a simply supported beam traversed by the ICE2 train traveling at different speeds in terms of acceleration (**a**) and strain (**b**) data estimated at mid-span.

To assess the impact of train speed on the AR coefficients, we conducted a parametric analysis as shown in Figure 4. This analysis includes six different train speeds: v=100, 150, 200, 250, 300, and 350 km/h. The results of these parametric analyses for both the acceleration and strain time series are presented in Figure 4a,b, respectively. Notably, when considering the acceleration time series, we observe greater variations in the amplitudes of the AR coefficients. In particular, we find mean relative variations of 18.37 for AR coefficients $a_{1}-a_{10}$ and a more substantial variation of 114.10% for AR coefficients $a_{11}-a_{26}$. The speed-induced variability is nearly halved when considering the strain time series, obtaining mean relative variations of 9.64 and 67.86% in the AR coefficients $a_{1}-a_{10}$ and $a_{11}-a_{26}$, respectively. From these analyses, it becomes evident that the variability of the AR coefficients (both in terms of acceleration and strain time series) is highly influenced by the train loading conditions. As a result, particular attention ought to be devoted to eliminating EOCs during the data normalization phase. Notably, these effects are considerably less pronounced when exploiting strain data, which supports the superior effectiveness of strain measurements for damage identification applications.

### 3.2. Case Study II: The Mascarat Bridge

#### 3.2.1. Description of the Structure

**Figure 5:**
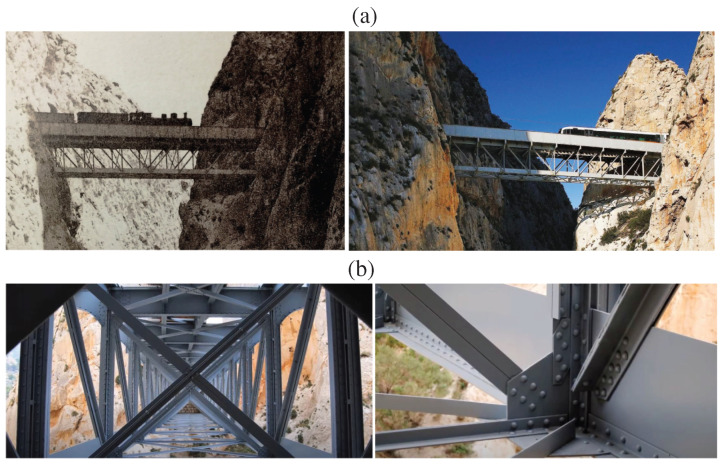
(**a**) Overview of the Mascarat Bridge. The left and right photographs depict the condition of the bridge in 1916 (Source: Photographic Archive of the Spanish Railway Foundation) and 2023 (Source: TodoAlicante), respectively. (**b**) Internal view of the viaduct and detail of the riveted joints.

The Mascarat Bridge stands as one of the most emblematic structures of the railway industrial heritage in the province of Alicante, Spain. Originally constructed in 1915 according to the design of the engineer José Carbonell (Figure 5a), the Mascarat Bridge remains operative in TRAM line 9 between the municipalities of Benidorm and Denia after undergoing restoration in 2016. Managed by Ferrocarrils de la Generalitat Valenciana (FGV), TRAM line 9 operates as a narrow-gauge, non-electrified line. This segment of the TRAM involves several steel truss viaducts built between 1913 and 1915 in accordance with the design regulations issued back in 1902. Specifically, the Mascarat Bridge is located along the Mascarat ravine between Altea and Calpe at PK 60 + 974. Spanning between two tunnels carved into the mountain at an altitude of approximately 140 m, the structure follows a single isostatic span with a total length of 43.20 m. Designed to accommodate diesel passenger trains and tipper trucks with a total tare weight of 66.24 tons, the bridge is constructed using rolled S355 steel forming beams of various formats (as detailed in Table 1) and joined together by hot rivets. The bridge comprises four main girders (paired on each side) forming a 4×4.2 m rectangular cross-section, connected by frames using Pratt-type triangulation (Figure 5b). The cross-section is further reinforced on the lower side through small beams forming cross braces and by heavy-duty beams serving as sleepers on the upper side. The 6 m wide upper deck supports a curved rail track with a radius of 175 m and inspection pathways. The bridge rests through neoprene supports atop abutments made of concrete plinths beneath the tunnel entrances.

**Figure 6:**
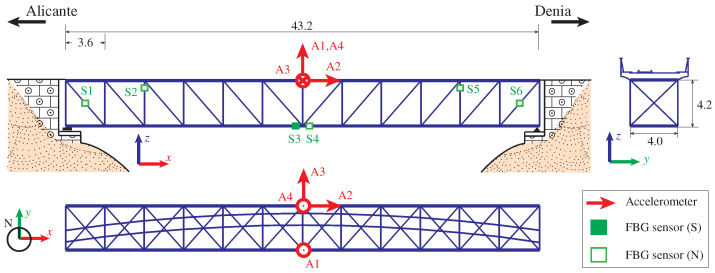
Sensors layout of the monitoring system installed in the Mascarat Bridge (units in m).

After the most recent restoration in 2016, the Department of Civil Engineering of the University of Alicante was commissioned to conduct a comprehensive series of structural performance tests on the bridge. These included both punctual and continuous static, semi-static, braking, and dynamic load testing. The monitoring system included a total of 29 sensors, of which only 9 were used in this work. The sensor layout, illustrated in Figure 6, comprises six point FBG fiber optic strain sensors (with dimensions of 195 × 15 × 5 mm and precision of 2 µϵ) and two accelerometers. The strain sensors (labeled from S1 to S6) are strategically located at two diagonal and vertical braces, as well as at mid-span of the lower girders. Moreover, accelerometers, two triaxial and one mono-axial (models B&K 4506-B-003 and B&K 4507-B-006, ±5 g, 490 mV/g), are located at mid-span of the viaduct (channels labeled from A1 to A4). In order to detect the train passages and trigger the data acquisition system, two photocells were positioned at the entrance and the exit of the viaduct. Strains and accelerations were recorded with a sampling frequency of 50 Hz. Furthermore, during night-time periods, 15 min long acquisitions of ambient accelerations were recorded at a sampling frequency of 200 Hz. Some of the monitoring recordings were retrieved in this study to evaluate the effectiveness of the proposed damage identification approach.

#### 3.2.2. Finite Element Model and Experimental Validation

**Figure 7:**
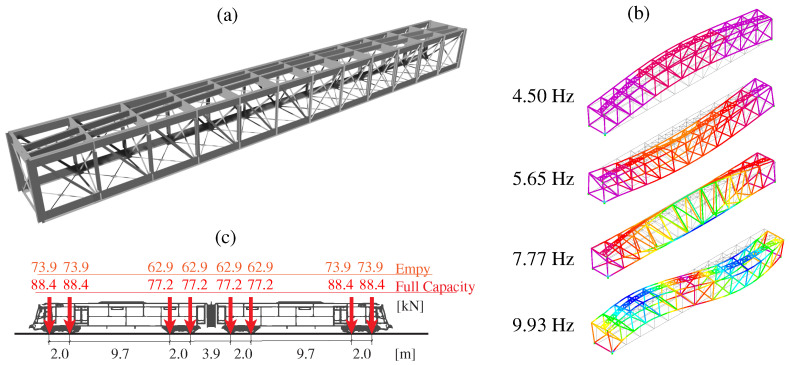
FEM of the Mascarat Bridge (**a**); first four numerical modal signatures of the FEM (**b**); and axle configuration of the 2500 Series Diesel Train (**c**).

With the aim of generating synthetic monitoring data and simulating damage scenarios to validate the proposed damage identification approach, a 3D FEM of the Mascarat Bridge has been developed in SAP2000, as shown in Figure 7a. The FEM is defined with two-node beam elements, with careful attention given to defining the steel profiles of the viaduct. This includes detailing the girders, cross-braces, joints, and connection plates. The material properties have been defined as elastic isotropic, with a Young’s modulus of 210 GPa, Poisson’s ratio of 0.3, and a mass density of 7850 kg/m${}^{3}$. Additional non-structural elements are incorporated in the form of distributed masses along the deck area. Given the rigid foundations on rock, the bridge is assumed to have simply supported conditions, thus neglecting any potential soil–structure interaction.

**Figure 8:**
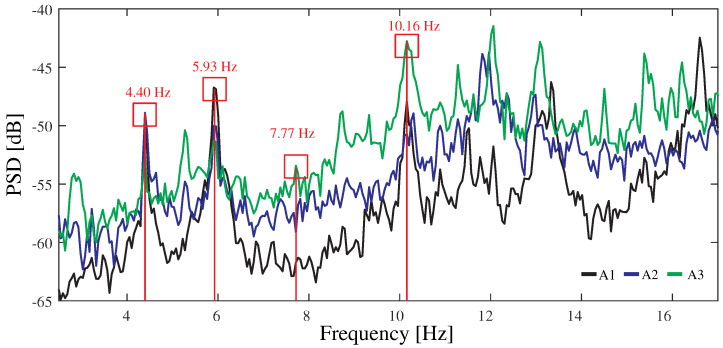
Power spectral density (PSD) functions of the ambient accelerations recorded by sensors A1 to A3 in the Mascarat Bridge (frequency resolution = 4.89 × 10${}^{-2}$ Hz).

The FEM has been calibrated to minimize discrepancies between experimentally identified resonant frequencies and the FEM predictions. To this end, the boundary conditions and the material properties have been adjusted by manual tuning. Figure 8 shows the power spectral density (PSD) functions obtained from a 5 min long ambient acceleration record acquired during night-time. The signals have been processed using the in-house software code P3P [60] with a filtering sequence involving elimination of abnormal peaks through Hanning windowing, moving mean detrend (time window = 0.08 s), and a high-pass Butterworth filter with cut-off frequency of 2 Hz. The frequency broadband of interest lies within 3 and 12 Hz, wherein some clear resonant peaks are observable. Specifically, four resonant peaks are identified at frequencies of 4.40, 5.93, 7.77, and 10.16 Hz. These frequencies are consistent with the outcomes of the linear modal analysis conducted on the FEM as reported in Figure 7b. These correspond to a first-order lateral bending mode, first-order vertical bending mode, first-order torsional mode, and second-order vertical bending mode. The average relative difference between the experimental and numerical resonant frequencies amounts to 2.27% (Table 2), demonstrating that the accuracy of the model suffices for the demonstrative purposes of this study.

**Figure 9:**
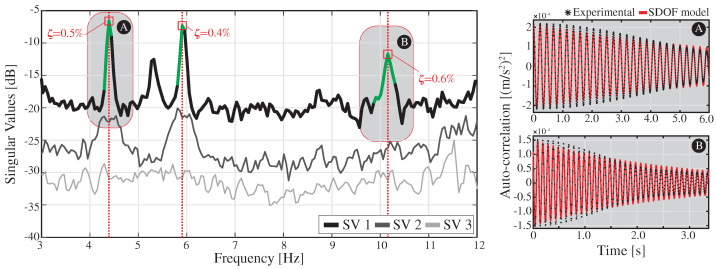
(**A**,**B**) Experimental estimation of the damping properties of the Mascarat Bridge using the EFDD method. The spectral bell identification is conducted considering the singular values fulfilling a modal assurance criterion (MAC) threshold of 0.8.

**Figure 10:**
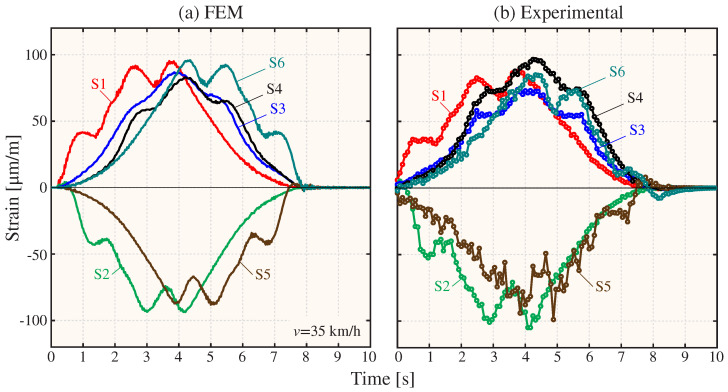
Comparison of numerical (**a**) and experimental (**b**) time series of deformation recorded in the Mascarat Bridge with the passage of the 2500 Series Diesel Train (fully loaded configuration) at 35 km/h.

The FEM has undergone further validation by examining the time series of strain resulting from the passage of the 2500 Series Diesel Train (fully loaded configuration) traveling at a speed of 35 km/h. The axle configurations for both fully loaded and empty conditions are presented in Figure 7b. For the sake of simplicity, the train load has been simulated as a tandem of moving masses traveling at a constant speed along the railway tracks, and the effects of the train–track–bridge interaction and rail irregularities are left for future work (refer to references [61,62] for a comprehensive state-of-the-art on these effects). It is important to note that such effects will contribute to the appearance of more fast-oscillating terms in the acceleration response, which would conceivably further motivate the use of strain data as discussed in the previous case study. Rayleigh damping has been defined considering the experimentally identified damping ratios for modes 1 and 3 (Figure 9) using the enhanced frequency domain decomposition (EFDD) method. To this end, the Ibrahim time domain (ITD) method has been employed on 20 cycles of the auto-correlation functions extracted using the natural excitation technique (NExT). The time history moving load simulation of the FEM was conducted using the Newmark-Beta direct integration approach with time steps of 10 ms. The analysis includes both the passage of the train and four times the fundamental period of the structure to analyze the free vibration response of the bridge. Throughout the simulations, accelerations and strains at nodes coinciding with the positions of the sensors are monitored (refer to Figure 7). The comparison between the analytical and experimental strain time series is presented in Figure 10. Notably, the FEM is capable of replicating the trend exhibited by all the strain sensors, achieving close fittings with most of the experimental curves. Only some significant differences are observed, primarily for sensors S3 and S4, which is conceivably attributable to the existence of semi-rigid connections between the steel members of the bridge. Nonetheless, an average absolute error of 10.34 µε is achieved. This level of accuracy is deemed sufficient for the aim of this study, and the incorporation of more sophisticated joint models is deferred for future work.

#### 3.2.3. Damage Identification Results

**Figure 11:**
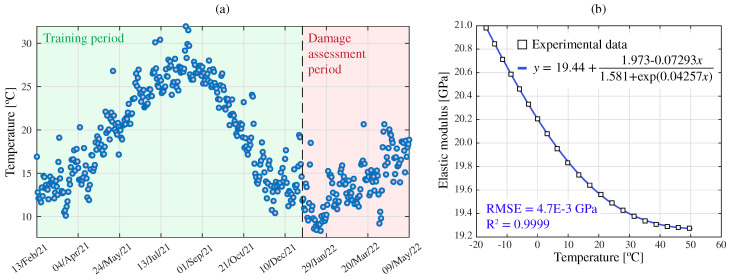
Time series of daily mean temperature in Alicante from 2021 until 2022 (Source: AEMET) (**a**), and temperature-dependent elastic properties of steel (experimental data taken from [63]) (**b**).

With the aim of validating the effectiveness of the proposed damage identification approach, an extensive synthetic dataset has been generated using the FEM of the Mascarat Bridge. To introduce realistic environmental effects into the simulations, the time series of mean daily temperature recorded in the province of Alicante from 2021 until 2022 have been considered as depicted in Figure 11a. The influence of these temperature values has been integrated in the FEM in the shape of temperature-dependent variations in the elastic modulus of steel. The experimental results reported by Meruane and Heylen [63] have been utilized for this purpose. After experimenting with various fitting functions, the best fit was achieved using the quotient between a first-order polynomial and an exponential term (refer to the insert in Figure 11b). The parameters of the fitting function were estimated through least-squares curve fitting, achieving a coefficient of determination of R${}^{2}=0.9999$ and a root mean squared error of RMSE = $4.7\times 10^{-3}$ GPa. On this basis, a training period of 1 year (365 samples) and a damage assessment period of 4.5 months (136 samples) have been defined. Additionally, the simulations have considered three different train speeds (defined according to the normal operation conditions of the bridge), namely 30, 50, and 80 km/h, as well as full and empty train configurations. This equates to a total of 3000 time history analyses. These simulations have been organized into datasets of 500 samples (16.5 months) by randomly selecting simulations associated with the different train configurations with equal probability. The primary goal of this dataset is to appraise the effectiveness of the statistical pattern recognition approach previously introduced in Section 2.2, which is intended to identify the variance in the AR coefficients induced by the (unmonitored) environmental temperature and train configuration. All the time series are recorded with a sampling frequency of 100 Hz and low-pass filtered with a cutoff frequency of 35 Hz. Additionally, the strain time series have been affected by a Gaussian white noise with a standard deviation of 8%, compatible with the noise levels observed in the experimental data.

**Figure 12:**
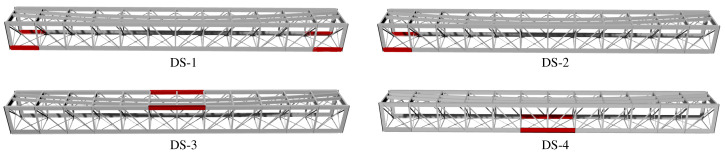
Steel members of the FEM of the Mascarat Bridge affected by the damage scenarios DS-*i*, i=1,…,4.

Four different damage scenarios have been included in the dataset by affecting the stiffness of certain structural members as shown in Figure 12. In particular, the analyses have considered those elements that have exhibited heightened susceptibility to fatigue damage according to the authors’ prior experience on the bridge. These include: DS-1—edges of the bottom girders; DS-2—bottom girders connected to the fixed support; DS-3—central section of the top girders; DS-4—central section of the top girders. Moreover, two different damage intensities *I* have been considered, defined as 20% and 40% reductions in the elastic modulus of the affected elements. The corresponding damage-induced variations in the resonant frequencies of the viaduct are reported in Table 2. The largest frequency decays are found for DS-1 (I=40%), with average and maximum decays of 0.8% and 1.2% (fundamental frequency), respectively. Therefore, the defined damage conditions can be assumed as moderate to mild, since such low-frequency decays are proximate to the minimum variations that are typically observable by modal-based damage identification techniques. In the synthetic dataset, the data samples between 400 and 449 correspond to the damage intensity I=20%, while the last 50 samples correspond to the intensity I=40%.

**Figure 13:**
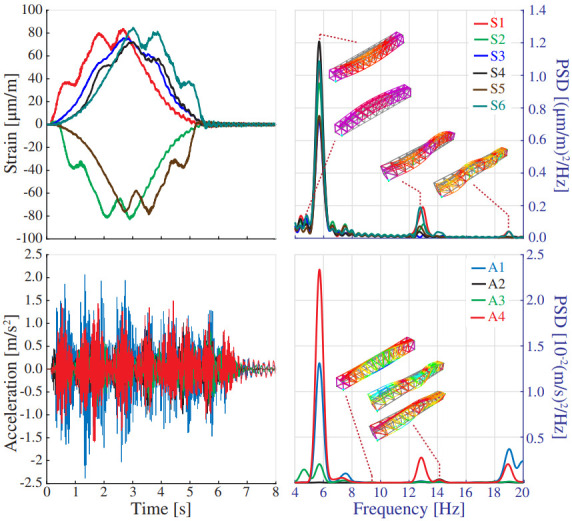
Time series of strain and acceleration and corresponding PSD functions with the passage of the 2500 Series Diesel Train (fully loaded configuration) at 50 km/h.

**Figure 14:**
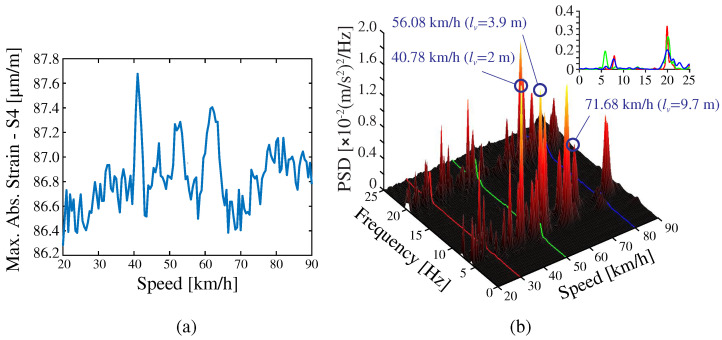
Maximum strain (S4) versus train speed (**a**); PSD of the acceleration recorded by sensor A1 for different train speeds (**b**) (2500 Series Diesel Train-fully loaded configuration).

Figure 13 provides one example of the numerical strain/acceleration time series along with the corresponding PSD curves obtained for the 2500 Series Diesel Train (fully loaded configuration) traveling at 50 km/h. It is evident in this figure that the acceleration time series is dominated by fast oscillating terms. Instead, the strain time series primarily exhibit low-oscillating components, which are less influenced by the dynamic amplification caused by the train’s motion. This trend is also reflected in the PSD curves, which reveal a higher presence of high-frequency modes in the acceleration data. In both cases, the train load predominantly excites the first-order vertical bending mode of the bridge. Nonetheless, the dynamic amplification phenomenon is highly influenced by the train’s speed. This can be noted in Figure 14a,b, which present the maximum strain recorded by S4 and the PSD of the accelerations recorded by A1 for train speeds ranging between 20 and 90 km/h. Is is extracted from this figure that operational effects induced by the train speed are notably lower when processing strain data (maximum dynamic amplification factor of 1.014). Nonetheless, when working with either strain or acceleration data, the frequency content in the structural response, and consequently the distribution of AR coefficients, is highly dependent on the train speed. Note in Figure 14b that, while the dynamic response is primarily dominated by the first-order vertical bending mode for the train speed of 50 km/h, the other two considered speeds of 30 and 80 km/h excite the first, second, and third vertical bending modes and the first-order torsional mode with roughly equal intensity. This behavior can be attributed to the train’s excitation frequency, estimated as $l_{v}/v$, where $l_{v}$ is the separation distance between the moving loads, and *v* is the train’s speed. Indeed, note in Figure 14b that, among the observable resonant peaks in the PSD surface, three correspond to the train speeds of 40.78, 56.08, and 71.68 km/h. These peaks are related to the critical train’s speeds extracted from $l_{v}=2.0$, 3.9 and 9.7 m, exciting, respectively, the first-order vertical bending, first-order torsional mode, and second-order vertical bending modes (refer to the train configuration in Figure 7c). Consequently, it can be concluded that the prevalence of the first-order vertical bending mode in Figure 13 is due to the proximity of the train’s speed (50 km/h) to the critical speed required for the bogies to excite this specific mode.

**Figure 15:**
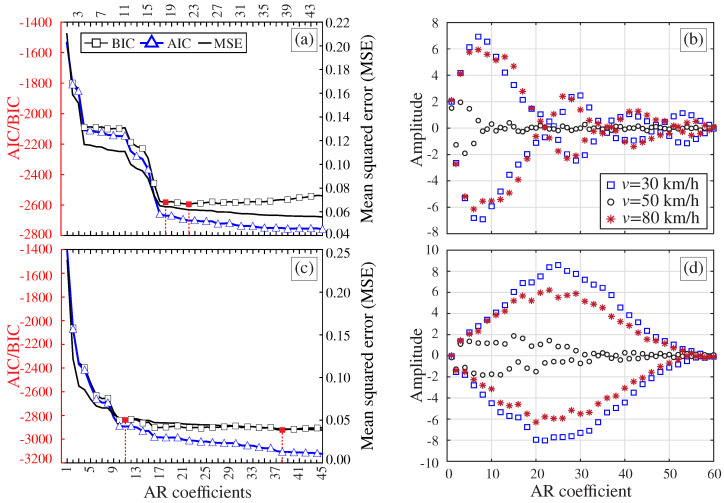
Convergence analyses of the AR coefficients extracted from the strain (S4) (**a**) and acceleration (A4) (**c**) time series for the Series 2500 (fully loaded configuration) traveling at 50 km/h. AR(60) coefficients extracted from the processing of strain (**b**) and acceleration (**d**) time series for different train speeds.

Figure 15a,c report the convergence analyses of the AR coefficients for the strain (S4) and acceleration (A4) time series from Figure 13, respectively. To eliminate the subjectivity in the selection of the model order, the optimal order is chosen according to the AIC, BIC, and RSS metrics previously introduced in Section 2.1. A total of 45 AR models with orders ranging from 1 to 45 have been constructed. It is observed in these figures that the minima of the BIC curve is found for the strain time series at m=22 (Figure 15a), while the minima for the acceleration time series is found at m=38 (Figure 15c). These results support the evidence from the analytical study in Section 3.1 on the possibility of utilizing more compact AR models when processing strain data. The effects of the train speed on the AR coefficients was further investigated, as shown in Figure 15b,d. In these figures, the recordings by sensors S4 and A4 are used to construct AR(60) models. Note that the amplitudes of the AR coefficients are concentrated within the first 10–20 terms when analyzing the strain series. Instead, the amplitudes when exploiting acceleration data increase almost monotonically until approximately $a_{25}$, followed by decreasing amplitudes until almost vanishing terms when approaching $a_{60}$. In both cases, the critical influence of the train speed is noticeable, leading to considerable variations in the AR coefficients. Note that the dynamic amplification phenomenon previously discussed in Figure 14 is also highlighted herein. It is clear that the time series for the train speed of 50 km/h requires a considerably lower number of AR coefficients, since the dynamic response is highly dominated by the first-order vertical bending mode. Finally, note that the train-induced effects are slightly larger when processing acceleration data. This is particularly evident when analyzing the results for the train speeds 30 km/h and 80 km/h, leading to maximum relative variations of about 17% and 33% when considering strain and acceleration data, respectively.

**Figure 16:**
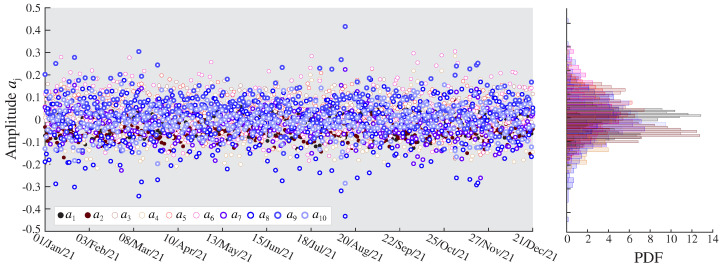
Time series of the ten first AR coefficients $a_{i}$, i=1,…,10, extracted during the training period (365 samples) and the corresponding probability density functions (PDFs).

**Figure 17:**
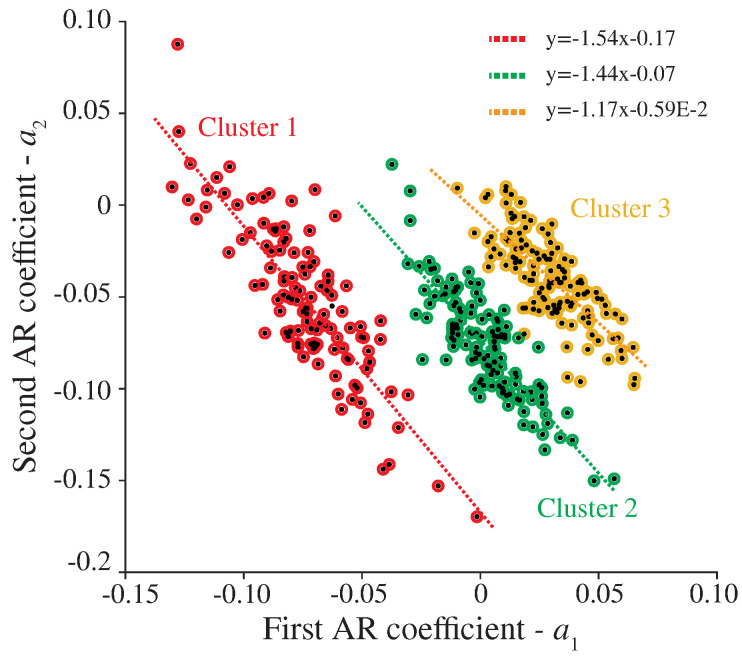
Example of the clustering results obtained for the first two AR coefficients ($a_{1}$ and $a_{2}$) during the training period (365 samples).

From the previous analyses, the proposed damage identification approach has been applied to the complete dataset. The optimal order of the AR models was automatically selected for all the sensors in the system by taking the minimum value of the BIC curves obtained for the first sample of the dataset and kept constant throughout the entire monitoring period. Figure 16 reports the time series of the first AR coefficients, $a_{i}$, i=1,…,10, extracted in the training period for sensor S1. Note that the dispersion originating from EOCs impedes the identification of any clear trend. This is also evidenced in the corresponding probability distribution functions (PDFs), which are multi-modal in many cases. Such a large dispersion will mask the appearance of damage. It is therefore of pivotal importance to adopt a robust statistical pattern recognition to identify the EOC-driven variability in the coefficients to attain efficient damage identification. A sample of the results of the clustering approach previously introduced in Section 2.2.1 is depicted in Figure 17 for sensor S1. Notably, the DBSCAN algorithm is capable of identifying three clear clusters corresponding to the three considered train speeds. Interestingly, the algorithm fails to identify different clusters corresponding to the two train load configurations. This variance in the AR coefficients along with the temperature-induced variations configure the dispersion of the identified clusters in Figure 17. These residual variances are further minimized by the cluster-wise PCA algorithm outlined above in Section 2.2.2. To this end, once all the data samples are organized into clusters, local PCA models are constructed by retaining the PCs, explaining up to 80% of the variance. The resulting residuals E are finally processed through Hotelling’s control charts to highlight the appearance of damage.

**Figure 18:**
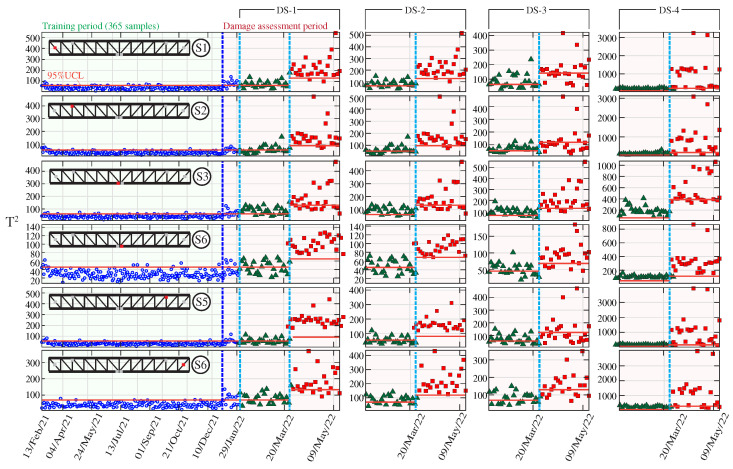
Hotelling’s control charts obtained for damage scenarios DS-1 to DS-4 when analyzing the strain recordings by sensors S1 to S6 in the Mascarat Bridge.

**Figure 19:**
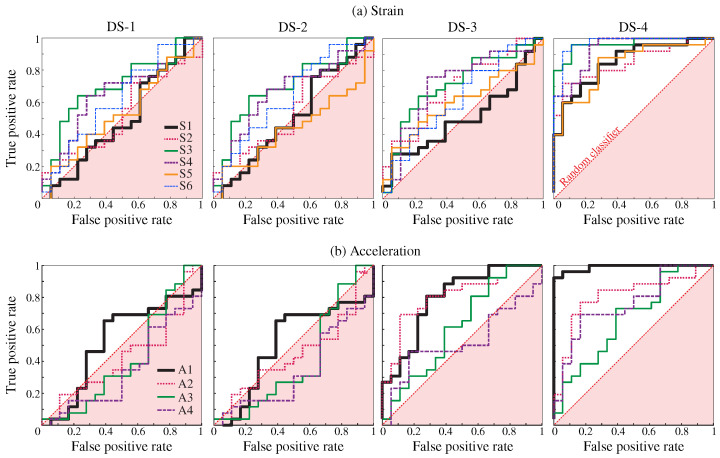
Receiver operating characteristic (ROC) curves obtained from Hotelling’s control charts defined in terms of AR strain (**a**) and acceleration (**b**) time series for low-intensity (I=20%) damage scenarios DS-*i*, i=1,…,4.

Figure 18 displays the resulting control charts obtained for the six strain sensors, S${}_{i}$, i=1,…,6, and the four considered damage scenarios, DS-*j*, j=1,…,4. In these analyses, a grouping size of r=2 has been selected, and the UCL has been established as the 95% confidence levels estimated in the training period (365 samples). It is important to remark that the UCL is re-estimated in the last damage step (I=40%) with the data belonging to the first damage step (I=20%). It can be noted in these figures that clear anomalies are detected every time a damage condition is introduced, finding accumulations of data points violating the UCL. Note that as the damage intensity increases, so does the nonconformity with respect to the training period. Therefore, these results demonstrate the potential of the developed approach not only for damage detection but also for providing some insight into the damage severity. Due to space constraints, the control charts corresponding to the AR coefficients extracted from the acceleration data are omitted. Instead, for the sake of reporting a more comprehensive comparison, the ROC curves extracted from the control charts obtained for the first damage intensity (I=20%) for the strain and acceleration time series of AR coefficients are presented in Figure 19a,b, respectively. This comparison clearly demonstrates the superior performance of strain data over acceleration data, the latter providing classifications in many cases below the random classifier (diagonal non-discriminating line) for most sensors and damage scenarios.

**Figure 20:**
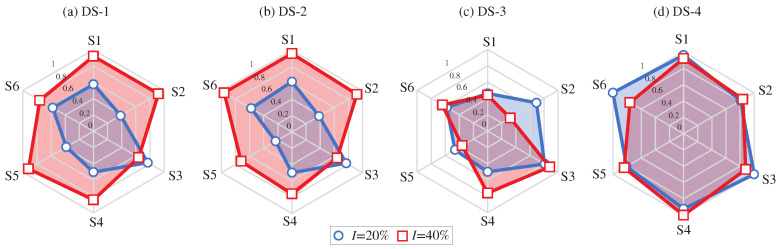
Sensor-level damage indices for damage scenarios DS-1 (**a**) to DS-4 (**d**) defined as the proportion of samples over the 95% confidence levels.

Finally, to provide a comprehensive metric for damage identification, a sensor-level damage index is defined as the proportion of samples violating the UCL (95%) for damage intensities I=20% and 40%. The results for damage scenarios DS-1 to DS-4 in the shape of radar graphs are depicted in Figure 20. It is interesting to note that the shape of the radar graphs significantly changes depending on the specific damage scenario. Specifically, note that the results for DS-1 exhibit a clear symmetry, while this symmetry is lost in DS-2. This is consistent with the actual location of the damage in DS-2 (refer to Figure 12), which is concentrated in S1 and S2. Furthermore, observe in DS-3 that the anomalies are concentrated in S3 and S4, which are the most proximate ones to the affected sections of the upper girders. Similarly, in DS-4, the anomalies are concentrated in S3 and S4, which are the sections directly affected by the damage scenario. Furthermore, important anomalies are found in S1 and S6, both located in two ending diagonals. From these results, it can be concluded that the interpretation of the sensor-based anomalies extracted from the AR strain coefficients also offers valuable information on the location of the damage, providing the developed damage identification approach with full damage identification (detection, quantification, and localization) capabilities.

## 4. Conclusions

This work has analyzed for the first time in the literature the use of AR modeling to continuously extract damage-sensitive features from strain data. To this end, a comprehensive SHM approach exploiting the continuous acquisition of strain data with the passage of trains has been developed. The proposed approach encompasses several key components: (i) automatic determination of the optimal model order through convergence analysis, (ii) long-term continuous extraction of AR coefficients as damage-sensitive features, (iii) and utilization of statistical pattern recognition and quality control charts for damage identification. For the minimization of benign fluctuations in the AR coefficients driven by EOCs, an innovative statistical pattern recognition approach combining DBSCAN and PCA has been developed. The effectiveness of the proposed approach has been validated through two case studies, namely a theoretical simply supported beam and a real-world in-operation railway bridge. For the latter case study, the Mascarat Viaduct in Alicante (Spain), a detailed three-dimensional FEM has been constructed and experimentally validated to generate an extensive synthetic monitoring dataset. The simulated database has considered the influence of environmental temperature through the definition of temperature-dependent material properties, as well as operational factors, namely the train’s speed and weight. Additionally, the developed FEM has been employed to simulate various damage scenarios. Overall, the reported numerical results have highlighted the superior performance of strain data over acceleration for achieving prompt damage identification. Indeed, the developed SHM approach has shown great potential for achieving full damage identification, including detection, quantification, and localization. The latter can be achieved by the interpretation of the sensor-level anomalies identified in the time series of AR coefficients, facilitating the construction of radar graphs to pinpoint damage locations. Key contributions of this study include:

- The presented SHM approach leverages continuous extraction of sensor-level AR coefficients, offering an easily implementable solution with minimal computational burden and expert intervention.
- Strain recordings under moving train load conditions are considerably less affected by high-oscillating components compared to acceleration data. This allows more compact AR models to be generated with AR coefficients less affected by operational factors (train speed and weight).
- To unveil the presence of damage, it is of pivotal importance to eliminate the effects of EOCs. To this end, the two-step statistical pattern recognition approach combining DBSCAN and PCA has shown proficiency in minimizing EOC-driven variance in the time series of continuously extracted AR coefficients.
- The proposed SHM approach provides a set of sensor-based control charts, offering great potential for damage detection, quantification, and localization. The latter can be achieved by interpreting radar graphs of the data points violating in-control upper control limits.

## Figures and Tables

**Table 1:** Steel profiles in the Mascarat Bridge.

Elements	Description
Connections	T-section formed by a 360 × 12 wing and a 560 × 11 web joined by 2 L 90 × 12. The upper and lower chords are reinforced by two 360 × 13 plates located on the wing in the central area of the bridge
Diagonals	L-type angles with widths between 80 and 120 mm and variable thickness
End uprights	L-type angle brackets with 70.9 × 90.7 mm dimensions and reinforcement plates of dimensions 360 × 90 mm, 160 × 90 mm and 150 × 11 mm
Remaining uprights	L-type angle brackets with 70 mm height and variable thickness, rein-forced with two 9 × 160 mm plates
Rafters	Double-T cross-sections with 450 × 9 mm and 70 × 9 mm flanges
Joists	Double-T cross-sections with 700 × 9 mm webs and 90 × 12 mm flanges

**Table 2:** Comparison of the experimentally identified resonant frequencies signatures of the Mascarat Bridge and the predictions of the FEM (DS-0). The effects of the damage scenarios DS-*i*, i=1,…,4, are reported in terms of relative frequency decays with respect to DS-0.

		DS-1 ($\Delta f_{i}/f_{i}$ [%])		DS-2 ($\Delta f_{i}/f_{i}$ [%])		DS-3 ($\Delta f_{i}/f_{i}$ [%])		DS-4 ($\Delta f_{i}/f_{i}$ [%])	
**Experim.**	**DS-0**	I=20 **%**	I=40 **%**	I=20 **%**	I=40 **%**	I=20 **%**	I=40 **%**	I=20 **%**	I=40 **%**
4.40	4.49	−0.50	−1.20	−0.49	−1.17	−0.18	−1.21	−0.23	−1.32
5.93	5.65	−0.67	−1.62	−0.26	−0.65	−0.53	−2.10	0.04	−0.72
7.77	7.77	−0.02	−0.06	0.00	−0.01	0.38	0.27	0.85	0.81
10.16	9.93	−0.13	−0.36	−0.12	−0.33	−0.05	−0.20	0.01	−0.04

## Appendix A. Closed-Form Solution of the Moving Load Problem for Simply Supported Beams

This appendix presents the derivation of the analytical solution to the moving load problem of a simply supported beam previously reported in Figure 2. Neglecting damping effects, the differential equation of vertical displacements u(x,τ) of an Euler–Bernoulli beam for a moving load reads:

(A1) $$\rho \left(x\right)\frac{\partial^{2}u\left(x,\tau \right)}{\partial \tau^{2}}+\frac{\partial^{2}}{\partial x^{2}}\left[EI\left(x\right)\frac{\partial^{2}u\left(x,\tau \right)}{\partial x^{2}}\right]+P\delta \left(x-v\tau \right)=0,$$

with τ=t−d/v being the relative time. Assuming that the system behaves linearly, the solution of Equation (A1) can be obtained by applying modal decomposition.

That is, u(x,τ)=Φ(x)y(τ), with Φ(x) being the modal matrix containing the mode shapes $\Phi_{n}\left(x\right)$ of the beam by columns, and y(τ) the vector of modal displacements. By virtue of the orthogonality property of the mode shapes, Equation (A1) can be decoupled into modal coordinates $y_{n}\left(\tau \right)$ as:

(A2) $$\int_{0}^{L}\Phi_{n}\left(x\right)\left[\rho \left(x\right)\frac{\partial^{2}y_{n}\left(\tau \right)}{\partial \tau^{2}}\right]\Phi_{n}\left(x\right)dx+\int_{0}^{L}\frac{\partial^{2}\Phi_{n}\left(x\right)}{\partial x^{2}}EI\left(x\right)\frac{\partial^{2}\Phi_{n}\left(x\right)}{\partial x^{2}}y_{n}\left(\tau \right)dx+P\Phi_{n}\left(v\tau \right)=0.$$

From Equation (A2), the generalized mass $M_{n}$ and stiffness $K_{n}$ values associated with the *n*-th mode can be identified as:

(A3) $$M_{n}=\int_{0}^{L}\Phi_{n}\left(x\right)\rho \left(x\right)\Phi_{n}\left(x\right)dx,\;K_{n}=\int_{0}^{L}\frac{\partial^{2}\Phi_{n}\left(x\right)}{\partial x^{2}}EI\left(x\right)\frac{\partial^{2}\Phi_{n}\left(x\right)}{\partial x^{2}}dx.$$

At this point, it is possible to include the damping effects by means of a modal damping ratio $\zeta_{n}$. Using dot notation to represent time derivatives, Equation (A2) can be rewritten in a more compact form as:

(A4) $${\ddot{y}}_{n}\left(\tau \right)+2\zeta_{n}\omega_{n}{\dot{y}}_{n}\left(\tau \right)+\omega_{n}^{2}y_{n}\left(y\right)+\left(P/M_{n}\right)\Phi_{n}\left(v\tau \right)=0.$$

where the term $\omega_{n}$ stands for the undamped angular frequency of the *n-th* mode, and $\zeta_{n}$ being is the corresponding modal damping ratio. The homogeneous solution of Equation (A4) can be readily derived as:

(A5) $$y_{n}^{h}\left(\tau \right)=e^{-\zeta_{n}\omega_{n}\tau }\left[A_{n}\cos\left(\omega_{n}^{d}\tau \right)+B_{n}\sin\left(\omega_{n}^{d}\tau \right)\right],$$

where terms $A_{n}$ and $B_{n}$ are constants to be determined by the boundary conditions. Terms $\omega_{n}$ and $\Phi_{n}$ denote the undamped angular frequency and mode shape of the *n*-th vibration mode and are given by:

(A6) $$\omega_{n}=n^{2}\pi^{2}\sqrt{\frac{EI}{\rho L^{4}}},\;\Phi_{n}\left(x\right)=\sin\left(\frac{n\pi x}{L}\right).$$

The resulting natural frequencies $f_{n}=\omega_{n}/2\pi$ and mode shapes of the first four vibration modes of the present case study are depicted in Figure 2b.

Defining the following non-dimensional parameters:

(A7) $$\Omega =n\pi \left(v\tau \right)/L,\;S_{n}=\Omega /\omega_{n},\;\eta =\frac{2PL^{2}}{EIn^{4}\pi^{4}}{\left[{\left(1-S_{n}^{2}\right)}^{2}+4{\left(\zeta_{n}S_{n}\right)}^{2}\right]}^{-1},$$

the particular solution of Equation (A1) reads:

(A8) $$y_{n}^{p}\left(\tau \right)=C_{n}\cos\left(\Omega \tau \right)+D_{n}\sin\left(\Omega \tau \right),$$

with $C_{n}$ and $D_{n}$ given by:

(A9) $$C_{n}=-2\zeta_{n}\eta L,\;D_{n}=\left(1-S_{n}^{2}\right)\eta L.$$

In this light, the solution of the modal coordinate $y_{n}\left(\tau \right)$ is obtained as $y_{n}\left(\tau \right)=y_{n}^{p}\left(\tau \right)+y_{n}^{h}\left(\tau \right)$, and terms $A_{n}$ and $B_{n}$ in Equation (A5) are found by applying the simply supported boundary conditions, which leads to:

(A10) $$A_{n}=-C_{n},\;B_{n}=\frac{\zeta_{n}\omega_{n}C_{n}+\omega_{n}^{d}}{\omega_{n}^{d}}.$$

Considering that the dynamic response is governed by *m* vibration modes, the solution reads:

(A11) $$u\left(x,\tau \right)=\sum_{n=1}^{m}\left\{e^{-\zeta_{n}\omega_{n}\tau }\left[A_{n}\cos\left(\omega_{n}^{d}\tau \right)+B_{n}\sin\left(\omega_{n}^{d}\tau \right)\right]+C_{n}\cos\left(\Omega \tau \right)+D_{n}\sin\left(\Omega \tau \right)\right\}\Phi_{n}\left(x\right)=Q\left(\tau \right)\;\Phi_{n}\left(x\right).$$

with $\omega_{n}^{d}=\omega_{n}\sqrt{1-\zeta_{n}^{2}}$ denoting the damped natural angular frequency. In this light, under the assumption of linear elasticity and Navier’s stress distribution for bending stresses, it is straightforward to extract the closed-form solution of the strain time-series at the top fiber of the cross-section as:

(A12) $$\varepsilon \left(x,\tau \right)=\frac{h_{s}}{2}Q\left(\tau \right)\;\frac{d^{2}\Phi_{n}\left(x\right)}{dx^{2}}.$$

Since the system is assumed to be linear, the previous formulation can be readily extended to general train compositions by means of linear superposition.
